# Influence of the Clinical Status on Stress Reticulocytes, CD 36 and CD 49d of SSFA_**2**_ Homozygous Sickle Cell Patients Followed in Abidjan

**DOI:** 10.1155/2014/273860

**Published:** 2014-02-27

**Authors:** Duni Sawadogo, Aïssata Tolo-Dilkébié, Mahawa Sangaré, Nelly Aguéhoundé, Hermance Kassi, Toussaint Latte

**Affiliations:** ^1^Department of Hematology, Faculty of Pharmacy, University Felix Houphouet Boigny, Cocody, BP 2308 Abidjan 08, Cote D'Ivoire; ^2^Unit of Hematology, Central Laboratory, Teaching Hospital of Yopougon, BP 632 Abidjan 21, Cote D'Ivoire; ^3^Clinic Hematology Service, Teaching Hospital of Yopougon, BP 632 Abidjan 21, Cote D'Ivoire; ^4^, , , , AIDS Biological Unit, Central Laboratory, Teaching Hospital of Yopougon, BP 632 Abidjan 21, Cote D'Ivoire

## Abstract

*Background and Objectives.* Interactions between sickle cells involving CD 49d, CD36, and the vascular endothelium may initiate vasoocclusion leading to acute painful episodes and multiple organ failure. *Materials and Methods.* We selected 60 SS patients who had never been treated by hydroxyurea. We performed a total blood count. We identified with immunophenotyping by flow cytometry total reticulocytes their distribution according to the degree of maturity (mature, intermediate, very immature) and CD 36^+^ and CD 49d^+^ antigens. Stress reticulocytes corresponded to the sum of intermediate and immature cells. *Results.* Subjects in crisis had more total reticulocytes and very immature reticulocytes than subjects in stationary phase (*P* < 0.05). During the crisis, total CD 36^+^ reticulocytes (214 870 ± 107 584/**μ**L versus 148 878 ± 115 024/**μ**L; *P* < 0.05) and the very immature CD 36^+^ reticulocytes (28.9 ± 7.9% versus 23.0 ± 6.4%; *P* < 0.05) increased. The clinical status had no impact on CD 49d^+^ reticulocytes. *Conclusion.* The rates of stress reticulocytes in general and those expressing CD 49d and CD 36 were very high. The clinical status had an influence on CD 36^+^ reticulocytes. The expression of adhesion molecules is only one of the parameters involved in sickle cell disease crisis.

## 1. Introduction

Sickle cell anemia (SCA) is a pathology characterized by acute pain and various organ failures, related to frequent vasoocclusive episodes. The mechanism that leads to these vasoocclusive events is not yet clearly defined. Vasoocclusion may be due to an interaction between sickle cells and the vascular endothelium. The result is a longer transit time of sickle cells in the capillary system [1–3]. Some of the molecules involved in these interactions have been identified for example CD 47, basal cell adhesion molecule-1/Lutheran (B-CAM-1/Lu), intercellular cell adhesion molecule 4 (ICAM-4) [3–5]. In this paper, we focused on *α*_4 _(CD 49d) *β*_1_ (CD 29) integrin or very late activation antigen (VLA) 4 and CD 36 which are, exclusively present on stress reticulocytes [1–5].

Hydroxyurea (HU) is a cancer chemotherapy agent that decreases the frequency of SCA crises. HU may exert its therapeutic effect by generating nitric oxide (anti-inflammatory) and increasing the level of antisickling fetal hemoglobin (Hb F) [3, 5, 6]. Higher levels of Hb F result in clinically milder sickle cell disease [3, 6]. HU also led to a significant decrease in the expression of the CD 36, CD 49d, and CD 29 genes [7].

Lee et al. [8] and Trinh-Trang-Tan et al. [9] found that the presence or absence of CD 36 had no effect on the clinical manifestations of SCA. Browne and Hebbel [2] and Styles et al. [3] had a completely different point of view.

In Côte d'Ivoire, the frequency of Hb S hovers around 14% with 2% of major forms [10]. HU is not part of the armamentarium used for the treatment of sickle cell patients. Patients exclusively treated in Cote d'Ivoire have never received HU. Maintenance treatment is based on folic acid and vasodilators [10]. Crises are treated by nonsteroidal anti-inflammatory drugs, particularly ketoprofen [10, 11].

It is not common to find studies with a population composed only of SS patients clearly showing the results of the subjects in steady state or in crisis. This is why we set a goal to investigate whether the clinical status, namely, the crisis or the steady state, had an impact on total reticulocytes, stress reticulocytes, and the expression of adhesion molecules and CD 36 and CD 49 d in SS black African patients never treated by HU in Abidjan.

## 2. Materials and Methods

This was a cross-sectional study carried out in the central laboratory and in the Department of Clinical Hematology of the University Hospital of Yopougon in Abidjan. The study population consisted of 60 SCA (Hb SS) patients who provided their informed consent for participation; of these 30 were in steady state and 30 were in crisis. Steady state was defined by a period of at least 1 month since the patient's last crisis [8, 12]. Patients had been exclusively treated in Cote d'Ivoire. They never had received HU. We did not include subjects who were transfused in the three months preceding the investigation. If the samples were hemolyzed, if they contained clot or if the immunophenotyping tests were not perform within 48 h, the patients were not also selected [8, 12]. The samples were taken as soon as the patients in crisis were admitted, the first day of the hospitalization.

Blood was collected by venipuncture performed at the elbow in a tube containing an anticoagulant, the Ethylendiaminetetraacetic acid (EDTA). We performed a complete blood count with Sysmex XT-2000i analyzer. The determination of the rate of reticulocytes and their degree of maturity was performed with immunophenotyping by flow cytometry with the FACSCalibur Flow Cytometer Becton Dickinson. We used the same device to demonstrate the CD 36 and CD 49d antigens on the surface of reticulocytes. At least 20 000 cells were analyzed on each samples. We used the following reagents: anti-CD 36 monoclonal antibodies coupled to fluorescein isothiocyanate (FITC), IgM isotope, kappa (FITC), anti-CD 49d monoclonal antibodies coupled to phycoerythrin (PE), IgG1 isotype, kappa (PE), Retic-Count or thiazole orange, saline phosphate, bovine serum albumin, and the fragment F (ab′) 2 IgG1 [3, 8].

The distribution of reticulocytes according to the degree of maturity with thiazole orange can be subdivided into mature reticulocytes (low fluorescence area or LFR), in reticulocytes of intermediate maturity (moderate fluorescence area or MFR) and in very immature reticulocytes (high fluorescence region or HFR). Stress reticulocytes correspond to the immature reticulocytes fraction (IRF) constituted by the sum of reticulocytes MFR and HFR [13]. The mean fluorescence intensity (MFI) was also measured.

VLA-4 is composed of CD 49d (*α*_4_ integrin) and CD 29 (*β*_1_ integrin). Both components were measured by Styles et al. [3] using flow cytometry. They found that that the expression of CD 49d and CD 29 were virtually identical. On this basis, we only sought the expression of CD 49d [3].

Data were compared using Student's *t*-test or Chi-Square test. A *P* value <0.05 indicated a significant difference.

## 3. Results

60 SS patients (32 male and 28 female) were studied. The average age was 15.12 ± 10.57 (2–43 years). We separated patients according to clinical status: crisis or steady state (Table 1). Patients in crisis or in steady state had a similar distribution with regard to age, sex ratio, and the Hb fractions (Table 1). On the other hand, in terms of clinical history, subjects in crisis had had a longer length of hospital stay and a higher number of transfusions (*P* < 0.05) than subjects in steady state. Clinical status had an impact on most elements of the complete blood count (Table 1). Severe anemia and leukocytosis with neutrophilia were the highlights of the complete blood count (Table 1).

Subjects in crisis had statistically significantly more total reticulocytes (absolute and relative values) and very immature reticulocytes, HFR, than subjects in steady state (Table 2).

All patients (60/60) had expressed CD 49d on the reticulocytes. The distribution according to the degree of maturity was similar during the crisis or the steady state. Very immature cells predominated (Table 3).

80% (48/60) of patients expressed the CD 36. The mean fluorescence intensity (MFI) for the stress reticulocytes was 120 ± 10 whereas for the more mature reticulocytes MFI it was lower (35 ± 7). Stress reticulocytes were strongly stained. The relative and absolute rates of CD 36^+^ reticulocytes were higher in crisis than in steady state (Table 4). Steady state was associated with an increase of CD 36^+^ intermediate maturity reticulocytes. Immature reticulocytes value was higher for the SS patients in crisis (Table 4).

## 4. Comments

Data on age and sex were similar to the other studies carried out in Abidjan which stressed that sickle cell patients were mostly teenagers or young male adults [10, 11].

Clinical history showed that patients who were recruited when they were in crisis received more transfusions and were hospitalized longer (Table 1). The frequency of painful crises requiring hospitalization and the length of hospital stay are clinical criteria used to assess the severity of SCA. Platt et al. [14] showed that the number of painful episodes in a year is a measure of the clinical severity of the disease and that it is associated with early death especially in patients over 20 years.

Indeed, SCA is associated with an abnormal inflammatory reaction which is even more important during crises [4, 10, 12]. Since the work of Sangare et al. [10] that has demonstrated the beneficial effect of nonsteroidal anti-inflammatory drugs, ketoprofen is used for treating seizures in Abidjan. It is associated with a vasodilator, pentoxifylline. This anti-inflammatory drug shortened significantly and without any side effects the duration of sickle cell crisis and had an effect greater than that of a major opioid analgesic. Patients never had been treated by HU.

The clinical status, namely, the vasoocclusive crisis, had an impact on almost all parameters of the complete blood count except for the level of platelets (Table 1). The complete blood count has demonstrated abnormalities commonly described as severe anemia and leukocytosis [3, 8, 10, 15]. In the patients followed in the present study, a higher leukocyte count in the peripheral circulation and infections were shown. Infections related to the increase of white blood cells were often associated with the occurrence of vasoocclusive episodes. Sluggish flow, increased transit time, hypoxia, and therefore sickling may be due to the probable interaction between sickle cells and leukocytes in the microcirculation [5]. A new multistep model for vasoocclusion in sickle disease is thus proposed. In this model, sickle cells or secondary inflammatory stimuli induced endothelial activation, leading to recruitment of adherent leukocytes. These leukocytes interacted with red blood sickle cells, thus hampering microvascular blood flow. In the end, sickle cells are trapped leading to vasoocclusion, as shown by Frenette [16].

The values of total reticulocytes (Table 2) were close to the results of other authors such as Styles et al. [3], Lee et al. [8], and Maier-Redelsperger et al. [15], which highlighted a hyperreticulocytosis ranging from 259 000/*μ*L [8] to 320 000/*μ*L [3, 15]. Crisis resulted in a statistically significant increase in relative and absolute values of total reticulocytes (Table 2). On the other hand the clinical status had no effect on the rate of stress reticulocytes (IFR) which was high. This result was surprising but it could be possible that some biological alterations still persisted at one month after the crisis even if the patients were in steady state.

Among the stress reticulocytes, the fraction of very immature cells was higher during crises than during steady state (10.7 ± 9.9% versus 6.7 ± 3.5%; *P* = 0.04). These values were lower than those of Maier-Redelsperger et al. [15] who found a frequency of 13% for very immature reticulocytes in SS patients [15]. Hebbel [1] was the first to show in 1980 that the red blood cells of SS patients had pathological adhesion to vascular endothelium. J. L. Wautier and M. P. Wautier [17] also noted that the adhesion was more important if the blood was collected in the vaso-occlusive crisis. According to Trinh-Trang-Tan et al. [9] reticulocytes which are more abundant and express more adhesion molecules may contribute to the hyperadhesiveness.

We only sought the expression of CD 49d which was positive for all patients (Table 2). The percentage of reticulocytes expressing CD 49d was 44.1 ± 17.7% for the painful crisis and 40.9 ± 7.7% for the steady state (Table 3). The difference was not significant (*P* = 0.37). Styles et al. [3] also found that the crisis or stationary phase did not influence the relative value of CD 49d^+^ reticulocytes. However, our results were higher than those of Styles et al. [3] and Lee et al. [8]. Styles et al. [3] had found that the percentage of reticulocytes expressing CD 49d was 29.0 ± 5.9%, that is, 129 000 ± 39 000/*μ*L. Lee et al. [8] showed that 22 ± 2% reticulocytes carried CD 49d. These high rates could be explained by the fact that the subjects we selected were much more anemic.

For CD 36^+^ subjects, the level of total reticulocytes was 53.4 ± 14.3% for subjects in crisis and 46.1 ± 11.4% for subjects in steady state (Table 4). These results differed from those of Lee et al. [8] who only found 24% ± 8.1% CD 36^+^ reticulocytes. They were close to those of Styles et al. [3] with 55.3 ± 6.4% CD 36^+^ reticulocytes before HU treatment. Browne and Hebbel [2] obtained 39.8 ± 21.9% for the total reticulocytes of CD 36^+^ patients. This value was lower than the results of Lee [8] but higher than the percentage given by Styles et al. [3].

We investigated SCA patients who never received HU. We compared the results with authors [3, 8] who had worked on SS patients treated by HU. It is well documented that HU decreases CD 49d and CD 36 expression even after months [3, 8]. In fact, Lee et al. [8] collected the samples before the introduction of HU treatment. Styles et al. [3] followed the SCA patients from the onset of therapy with HU.

According to Styles et al. [3], we found that CD 36 expression on reticulocytes was higher than that of CD 49d. Nevertheless, these authors emphasized that adhesion mediated by VLA-4 or *α*_4_ (CD 49d) *β*_1_ (CD 29) integrin would be more tenacious than that involving the CD 36 [3].

The contribution of CD 36 has been called into question with the finding that sickle cell patients who have a CD 36 deficiency of reticulocytes and mature red blood cells can have a normal clinical course [8, 9]. Trinh-Trang-Tan et al. [9] showed that there is dissociation between adhesiveness and adhesion molecules. It is therefore conceivable that CD 36, although in reduced amounts, might be activated by abnormal constitutive phosphorylation [9].

Blood flow is compromised in sickle microcirculation. Under low flow, sickle cell adherence to endothelium increased with contact time in the absence of endothelial activation or adhesive protein addition. Contact time between sickle cells and endothelium seemed a more important determinant of adherence than high-affinity receptor-ligand interactions [18].

The signalization cascade leading to receptor activation rather than the expression level only of adhesion molecules should also play an important role in the adhesion of sickle cells to the blood vessels [4].

## 5. Conclusion

In SCA, the clinical status—crisis or steady state—had an impact on the clinical history, namely, the number of hospital days and the number of transfusions. Anemia, leukocytosis, and total and immature reticulocytes were higher in patients in crisis than those in steady state. There was no difference between subjects in crisis and those in steady state for CD 49d^+^ reticulocytes. Concerning the CD 36^+^ subjects, the crisis had resulted in an increase in total reticulocytes and very immature reticulocytes.

SCA is the result of complex mechanism involving adhesion molecules such as CD 49d and CD 36. There is a correlation between the adhesion systems and the stress reticulocytes. However these facts are not sufficient to predict the occurrence and severity of sickle cell crisis.

## Figures and Tables

**Table 1:** Influence of clinical status on epidemiological, clinical, and biological parameters.

Parameters	Painful crisis (*n* = 30)	Steady state (*n* = 30)	*P*
	m ± sd (min–max)	m ± sd (min–max)	
Age	13.9 ± 9 (2–37)	16.4 ± 11.9 (2–43)	0.364
Sex (M/F)	1.14	1.14	0.795
Number of crises/year	2.5 ± 1.01 (1–4)	2.3 ± 0.84 (1–4)	0.406
Hospitalization days/year	1.4 ± 1.01 (0–6)	0.47 ± 0.63 (0–2)	0.0002
Transfusions/year	0.93 ± 0.52 (0–2)	0.43 ± 0.5 (0-1)	0.0004
Hb S (%)	86.8 ± 5.7 (76–95)	86.1 ± 4.5 (73.6–93.6)	0.622
Hb F (%)	18.8 ± 5.1 (2.5–23.2)	11.5 ± 4.4 (3.9–23.6)	0.595
Hb A_2_ (%)	2.3 ± 1.01 (1–4)	2.4 ± 0.6 (1.5–4.2)	0.599
Red blood cells/*μ*L	2 011 000 ± 698 000	2 593 000 ± 605 000	0.001
Hemoglobin (g/dL)	5.64 ± 1.81	6.98 ± 1.47	0.0024
Hematocrit (%)	17.83 ± 5.12	21.01 ± 4.03	0.01
Mean cell volume (fL)	91.02 ± 10.94	82.42 ± 11.09	0.004
MCH (pg)	28.43 ± 3.15	27.32 ± 3.84	0.224
MCHC (%)	31.32 ± 2.22	33.15 ± 1.57	0.0005
Leukocytes/*μ*L	23 778 ± 13 038	14 206 ± 11 515	0.004
Neutrophils/*μ*L	13 046 ± 6 685	5 838 ± 3 013	10^−6^
Platelets/*μ*L	323 133 ± 159 263	407 867 ± 173 572	0.53

m ± sd (min–max): mean ± standard deviation (minimum–maximum).

MCV: mean cell volume.

MVH: mean cell hemoglobin.

MCHC: mean cell hemoglobin concentration.

*P*: Student's *t*-test.

**Table 2:** Distribution of reticulocytes according to their degree of maturity and to the clinical status.

Parameters	Painful crisis (*n* = 30)	Steady state (*n* = 30)	*P*
	m ± sd	m ± sd	
Total reticulocytes (%)	15.5 ± 10	8.4 ± 4.9	0.003
Total reticulocytes (/*μ*L)	283 678 ± 153 711	200 721 ± 10 708	0.002
LFR	51.5 ± 15.9	56.6 ± 7.5	0.3
MFR	37.9 ± 10	36.7 ± 5.4	0.18
HFR (%)	10.7 ± 9.9	6.7 ± 3.5	0.012
IRF (%)	48.6 ± 15.9	43.4 ± 7.5	0.3

m ± sd: mean ± standard deviation.

*P*: Chi-Square test.

LFR: low fluorescence reticulocytes or mature reticulocytes.

MFR: medium fluorescence reticulocytes or semimature reticulocytes.

HFR: high fluorescence reticulocytes or immature reticulocytes.

IRF: index reticulocytes fraction or stress reticulocytes (MFR + HFR).

**Table 3:** Profile of CD 49d^+^ reticulocytes according to their degree of maturity and the clinical status.

CD 49d^+^ patients			
Parameters	Painful crisis (*n* = 30)	Steady state (*n* = 30)	*P*
	m ± sd	m ± sd	
Total reticulocytes (%)	44.1 ± 17.7	40.9 ± 7.7	0.13
Total reticulocytes (/*μ*L)	134 604 ± 108 223	83 782 ± 54 222	0.06
LFR	9.9 ± 19.6	5.8 ± 2.1	0.91
MFR	26.4 ± 8.5	28.4 ± 6.5	0.8
HFR (%)	63.7 ± 18.1	65.8 ± 7.6	0.19
IRF (%)	90.1 ± 19.6	94.2 ± 2.1	0.65

m ± sd: mean ± standard deviation.

*P*: Chi-Square test.

LFR: low fluorescence reticulocytes or mature reticulocytes.

MFR: medium fluorescence reticulocytes or semimature reticulocytes.

HFR: high fluorescence reticulocytes or immature reticulocytes.

IRF: index reticulocytes fraction or stress reticulocytes (MFR + HFR).

**Table 4:** Distribution of CD 36^+^ reticulocytes according to their degree of maturity and the clinical status.

CD 36^+^ patients			
Parameters	Painful crisis (*n* = 23)	Steady state (*n* = 25)	*P*
	m ± sd	m ± sd	
Total reticulocytes (%)	53.4 ± 14.3	46.1 ± 11.4	0.006
Total reticulocytes (/*μ*L)	214 870 ± 107 584	144 878 ± 115 024	10^−5^
LFR	9.2 ± 7.4	8.9 ± 6.4	0.32
MFR	61.9 ± 8.9	68.1 ± 6.2	0.001
HFR (%)	28.9 ± 7.9	23 ± 6.4	0.002
IRF (%)	90.8 ± 7.4	91.2 ± 6.4	0.3

m ± sd: mean ± standard deviation.

*P*: Chi-Square test.

LFR: low fluorescence reticulocytes or mature reticulocytes.

MFR: medium fluorescence reticulocytes or semimature reticulocytes.

HFR: high fluorescence reticulocytes or immature reticulocytes.

IRF: index reticulocytes fraction or stress reticulocytes (MFR + HFR).
